# In Vitro Spermatogenesis by Three-dimensional Culture of Spermatogonial Stem Cells on Decellularized Testicular Matrix

**DOI:** 10.31661/gmj.v8i0.1565

**Published:** 2019-12-15

**Authors:** Sepideh Ashouri Movassagh, Mehdi Banitalebi Dehkordi, Morteza Koruji, Gholamreza Pourmand, Parvaneh Farzaneh, Sanaz Ashouri Movassagh, Ayob Jabari, Azam Samadian, Farnaz Khadivi, Mehdi Abbasi

**Affiliations:** ^1^Department of Anatomy, School of Medicine, Tehran University of Medical Sciences, Tehran, Iran; ^2^Human and Animal Cell Bank, Iranian Biological Resource Center (IBRC), ACECR, Tehran, Iran; ^3^Cellular and Molecular Research Center, Basic Health Sciences Institute, Shahrekord University of Medical Science, Shahrekord, Iran; ^4^Cellular and Molecular Research Center & Department of Anatomical Sciences, Iran University of Medical Sciences, Tehran, Iran; ^5^Urology Research Center, Sina Hospital, TehranUniversity of Medical Sciences, Tehran, Iran; ^6^Midwifery and Disease Reproduction group, College of Veterinary Medicine, Islamic Azad University, Science and Research Unite, Tehran, Iran; ^7^Department of Stem Cells and Developmental Biology, Cell Science Research Center, Royan Institute for Stem Cell Biology and Technology, ACECR, Tehran, Iran

**Keywords:** Spermatogonial Stem Cells, Decellularization, Testicular Matrix, Proliferation, Diffetentiation

## Abstract

**Background:**

In the males, Spermatogonial Stem Cells (SSCs) contribute to the production of sex cells and fertility. *In vitro* SSCs culture can operate as an effective strategy for studies on spermatogenesis and male infertility treatment. Cell culture in a three-dimensional (3D) substrate, relative to a two-dimensional substrate (2D), creates better conditions for cell interaction and is closer to *in vivo* conditions. In the present study, in order to create a 3D matrix substrate, decellularized testicular matrix (DTM) was used to engender optimal conditions for SSCs culture and differentiation.

**Materials and Methods:**

After, testicular cells enzymatic extraction from testes of brain-dead donors, the SSCs were proliferated in a specific culture medium for four weeks, and after confirming the identity of the colonies derived from the growth of these cells, they were cultured on a layer of DTM as well as in 2D condition with a differentiated culture medium. In the Sixth week since the initiation of the differentiation culture, the expression of pre meiotic (*OCT4 & PLZF* ), meiotic (*SCP3 & BOULE*) and post meiotic (*CREM & Protamine-2*) genes were measured in both groups.

**Results:**

The results indicated that the expression of pre meiotic, meiotic and post meiotic genes was significantly higher in the cells cultured on DTM (P ≤ 0.001).

**Conclusion:**

SSCs culture in DTM with the creation of ECM and similar conditions with in vivo can be regarded as a way of demonstrating spermatogenesis in vitro, which can be adopted as a treatment modality for male infertility.

## Introduction

SSCs are located on the basement membrane of seminiferous tubules, which can contribute to male fertility by proliferating and differentiating themselves into sperm cells [1]. Therefore, in order to resolve the fertility problem in a male group afflicted with meiotic division arrested or children undergoing radiotherapy and chemotherapy due to cancer and susceptible to potential male fertility driven by the loss of SSCs, *in vitro* SSC proliferation and differentiation can be promising for treating male infertility [2]. Meiosis division is considered as one of the problems of SSCs culture in the laboratory. Accordingly, extensive studies have been performed to create ideal culture conditions for SSC proliferation and differentiation. Given that the cells lie in a three-dimensional microenvironment with complex cell-cell and cell-matrix inter-relationships in the living tissue, [3-9], the present study aimed to prepare a 3D substrate benefitting from biocompatibility, which can create the same conditions as *in vivo* for the cell. The cells in the body are in contact with the extracellular matrix (ECM), which prepares the proper conditions for cellular communication and includes various types of collagen, proteoglycan, glycoprotein, and glycosaminoglycan compounds varying in size in different tissues. It is challenging to generate a substrate acting similarly as ECM, but the application of a substrate provided from decellularized tissue alongside ECM preservation can effectively contribute to the improvement of culture conditions *in vitro*. A large number of studies have focused on cell culture and differentiation in decellularized tissues. [10-12]. In the present research, given the specific texture of ECM in testicular tissue, a substrate generated from sheep testicular decellularization was used to improve SSC culture and differentiation conditions.

## Materials and methods

### Preparation of testicular tissue

Testes were obtained from five brain-dead donors with history of fertility. The tissues were taken from Organ Procurement Unit (OPU) in Sina Hospital affiliated with Tehran University of Medical Sciences. This is done after transferring the patients to the donation unit and after having received the written consent of the family of each donor for the use of the testicular tissue for research propose. Ethics Committee of Tehran University of Medical Sciences (IR.TUMS.REC1394.1751) confirmed the project. Following autopsy, the testes were isolated and transferred to a cell culture laboratory in a culture medium containing antibiotic at 4 °C.

### Testicular cell isolation

After autopsy, the testicular tissues were removed and transferred to the culture lab in a culture medium containing antibiotic at 4 °C. In the laboratory, after the testes were washed with PBS, the testicular capsule was removed, and then divided into smaller fragments by mechanical means. Then, based on the existing protocols and with slight modification, the testicular tissue cells were isolated by enzymatic method [13, 14]. Accordingly, the testicular fragments were first placed in the solution containing collagenase Type I 1 mg/ml (Sigma,St louis, MO) and DNase 0.5 mg/ml ((Thermo Fisher Scientific, USA) in a 37 °C shaker incubator for 10 min. After centrifugation and washing, the tissues were again placed in the solution containing collagenase 1 mg/ml, DNase 0.5 mg/ml, and hyaluronidase mg/m 1.5 (Sigma,St louis, MO) in a shaker incubator at 37 °C for 10 min. In addition, the digested tissues were transferred from the cell strainer 70μm, and accordingly the cells were washed and cultured.

### Culture and proliferation of SSCs

After counting and evaluating the percentage of viability of the cells using trypan blue solution 0.4%, the cells were cultured in DMEM/F12 culture medium (Gibco, USA) containing 10% FBS (Gibco, USA) with 100 U/ml penicillin and 100 μg/ml streptomycin (both from Gibco, USA). SSCs are lower in number than somatic cells, which are attached to the culture dish after some delay. In order to isolate these cells further, the supernatant was removed after 24 h since the initial culture, and then centrifugation was performed to isolate the cells which were not yet attached to the substrate. Following the centrifugation, for the more specific growth of SSCs, the cells were cultured in the culture dish coated with 0.2% gelatin and culture medium DMEM/F12 containing 5% Knockout serum replacement (KSR) (Invitrogen, USA) and growth factors including 10 ng/ml GDNF, 103 U/ml LIF, 10 ng/ml FGF, and 20 ng/ml EGF. All growth factors were purchased from Sigma, Germany. Then, the cells were incubated at 37 °C and 5% CO2.

### Preparation of the decellularized matrix from sheep testicles

Sheep testicles were transferred from slaughterhouse to a lab in a PBS solution at 37 °C. Following the transfer, the tissues were washed several times in PBS solution, and accordingly they were placed in a freezer for 24 h, and the sections with a diameter of 100 μm were dissected after freezing. 1% sodium dodecyl sulfate (SDS) as the solution was used for decellularization, and the sections were immersed in for 24 h. Further, they were placed in a phosphate saline buffer for 2 h to remove detergents. Finally, the decellularized tissue sections were sterilized in ethanol 70% for one hour, and immersed in PBS for 2 h after washing.

### DNA content analysis

After the testicular tissue decellularization, the DNA was extracted using QIAamp DNA mini kit (Qiagen) in accordance with the manufacturing company instructions in order to ensure the absence of the cells in the tissue. The purity and concentration of DNA were determined using the ND-3800 spectrophotometer (Nano-drop Technologies, Hercuvan, Malaysia).

### Tissue staining and examination with optical microscopy

A histological examination was performed to verify the tissue quality and decellularization of DTM and an analysis of the migration of cells in to DTM after recellularization. For this purpose, the tissues were fixed at 4% paraformaldehyde. The tissues were placed in paraffin and molded after alcohol dehydration and immersion in xylol solution. The sections of 5 μm thickness were dissected from the tissues by using microtome, and the sections were stained via hematoxylin -eosin (H & E) following the removal of the paraffin.

### Tissue examination with electron microscope

After fixing the DTM in 2.5% glutaraldehyde, it was washed with PBS and fixed in osmium tetroxide for 2 h. After dewatering with the increasing degrees of ethanol, the tissues were covered with gold-palladium coating, and then the tissue structure was examined via an electron microscope following decellularization and recellularization.

### SCCs culture and differentiation on DTM

In order to evaluate the effect of DTM on the SCCs culture and differentiation, after four weeks since the initiation of SCCs culture and proliferation, 1.5×105 cells of the SSCs were cultured in 24-well plates with a DTM layer placed at the bottom of each well and in a 24-well plate in the absence of DTM in a differentiation medium whose base was DMEM/F12 (Gibco, USA) medium supplemented with 5% KSR, 5% fetal bovine serum (FBS) (Gibco, USA), 10 µg/ml insulin-transferrin-selenium solution (Gibco, USA), 3.3×10-7 M retinoic acid (Sigma, Germany), 10 µg/ml vitamin E (Gibco, USA), 10-4 M vitamin C (Sigma, Germany), 1 m M pyruvate (Sigma, Germany), 2.5×10-5 U human FSH (Merk, Germany), 10-7 M testosteron (Sigma, Germany), 1X antibiotic-antimycotic solution (Gibco, USA) [15]. Finally, the culture was performed in sixth week intervals under both conditions.

### Immunocytochemistry (ICC)

To confirm the identity of the colonies derived from the growth of SSCs, these cells were examined with GFRα1 (Thermo Scientific) and PLZF (Santa cruz) prior to the differentiation culture. In contrast, following the differentiation culture, the cells were examined with the antibodies BOULE (Biorbyt) and SCP-3 (Abcam) for spermatocytes, and the antibodies protamine-2 (Biorbyt) and CREM (Biorbyt) for spermatids via an immunocytochemistry method. Further, 0.3% Triton solution (Sigma, Germany) was used for the permeability of the membrane of the cells after fixing the cells in paraforldehide 4% in 4 0C for 24 h. Furthermore, 10% goat serum (Sigma, Germany) was added after washing with PBS. Then, the cells were incubated for 1 night in the presence of initial antibody at 4 °C. The cells were then washed, and incubated in the presence of secondary antibody for one hour and a half at 37 °C in darkness. Afterwards, the cells were washed, DAPI was added, and the cells were observed by an Olympus fluorescence microscope by the lens 400 for confirmation of markers.

### Real-time PCR

After terminating the sixth week of the differentiation culture, the RNA of the cells cultured on DTM and 2D substrate was extracted by using the Trizol reagent kit (ready Mini KIT, Qiagen, USA) and the manufacturer’s instruction. Purity and concentration of RNA was determined using ND-3800 spectrophotometer (Nano-drop Technologies, Hercuvan, Malaysia). The relative expression levels of per meiotic (*OCT4 & PLZF*), meiotic (*SCP3 & BOULE*), and post meiotic (*CREM & Protamine-2*) genes were measured by real-time PCR (qPCR), The specific primers are listed in Table-1. Total RNA (2 µg) was applied for cDNA using a Prime Script RT reagent kit (Takara Bio Inc, Tokyo, Japan) according to the manufacturer’s instructions. Real-time PCR was performed using a qPCR machine (Applied Bio Systems, Foster City, USA) and the SYBR Premix Ex Taq Kit (Tli RNaseH Plus). The qPCR steps were as follows: initial denaturation at 95˚C for 30 s, amplification for 40 cycles of denaturation at 95˚C for 5 s, annealing at 60˚C for 20 s, melting curve analysis at 95 ˚C for 15s, 60 ˚C for 1 min, 95 ˚C for 15s. All samples were normalized against glyceraldehyde-3-phosphate dehydrogenase (GAPDH) as an internal control, and the relative quantification of gene expression was determined using the comparative CT method (ΔΔCT).

### Statistical Analysis

ANOVA was used for data analysis in SPSS 10.0 (SPSS Inc., Chicago, IL).The P-value of <0.05 was considered as statistically significant.

## Results

### Characterization of SSCs colonies

**Figure 1:**
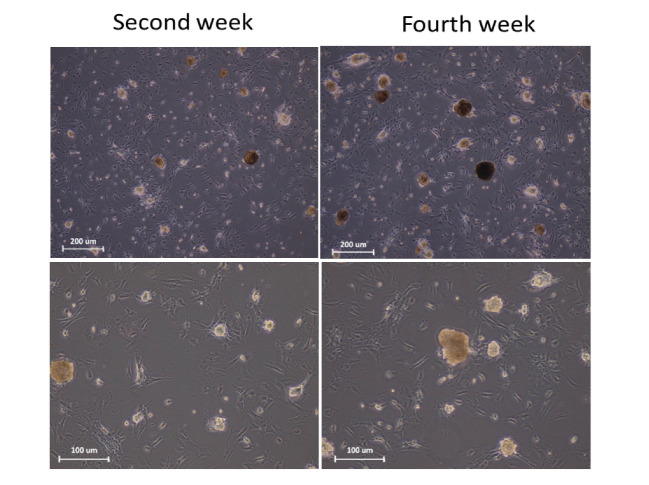
Microscopic morphology of SSCs colonies cultured in gelatin coated plate at the end of the second and fourth week. The sizes, diameter and number of SSCs colonies increased after fourth week (A). Scale bars=200 μm and 100 μm.

**Figure 2:**
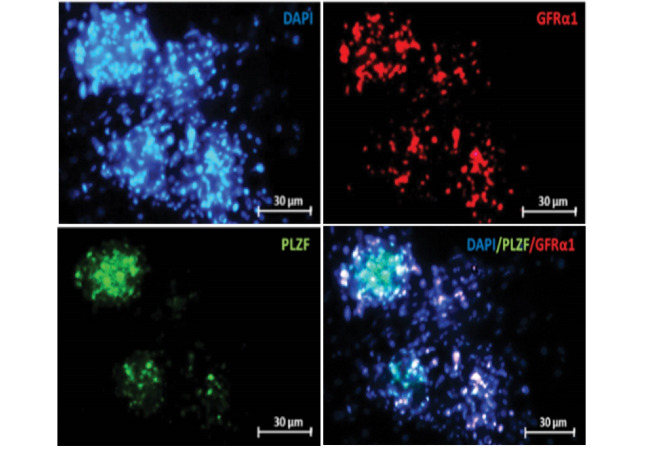
Immunocytochemistry staining of the colonies were derived from SSCs proliferation to evaluate the expression of spermatogonial markers (*GFRα1 & PLZF*) with their specific antibodies. Scale bars=30 μm.

The colonies derived from SSCs proliferation were observed approximately two weeks after the culture of the cells isolated from the testicular tissue by enzymatic digestion (Figure-1). After four weeks since the inception of the culture and proliferation of the cells, the colonies formed were examined using PLZF & GFRα1 antibodies via immunocytochemistry. Based on the results, the majority of the colonies could express both of the markers (Figure-2).

### DNA contend analysis

**Figure 3:**
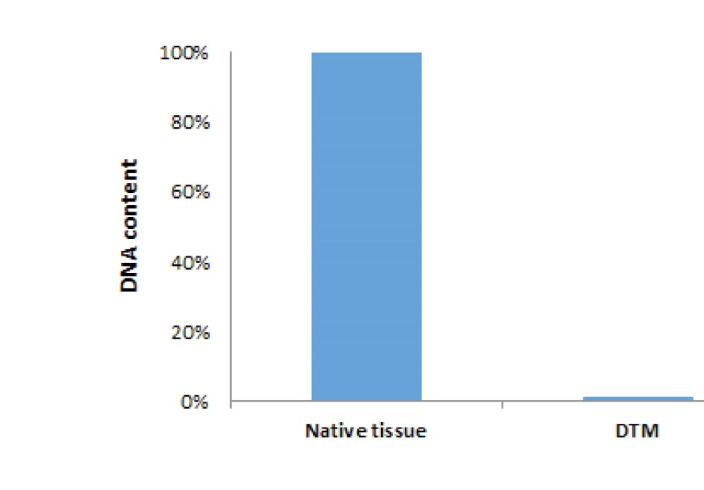
Quantification of DNA in native tissue and DTM Showed a significant decrease and almost complete loss of the DNA content in DTM. (P=0.001)

After assessing, the quantitation of the DNA was extracted from DTM, and native testicular tissue showed that almost the entire DNA was removed from DTM, and its DNA related to the testicular tissue was found to be 1 ± 1%.(P =0.001, Figure-3).

### Evaluation of the quality of ECM structure in DTM

**Figure 4:**
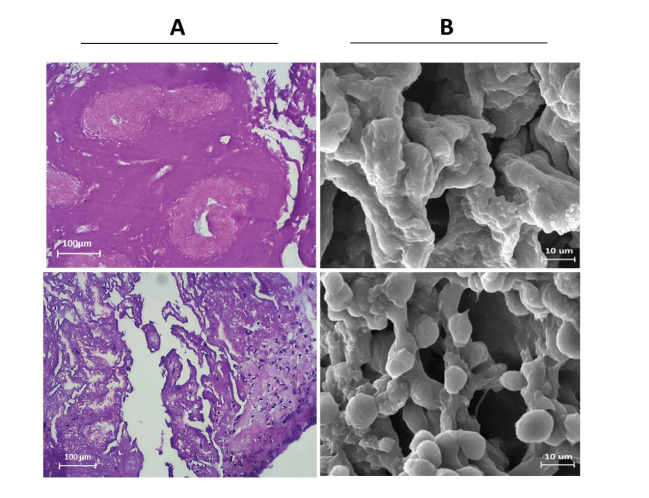
Histological staining of DTM via H and E after decellularization and recellularization **(A)**, Scale bars =100 μm. Scaning electron microscopy for evaluation of the 3D structure of the DTM following decellularization and recellularization** (B)**. Scale bars=10 μm.

In order to evaluate the ECM quality in decellularized tissue, the DTM was stained using H & E staining. The results indicated that the matrix structure and seminiferous tubules of the testicular tissue were properly preserved while the tissue was depleted from the cell (Figure-4). The assessment of the three-dimensional structure of the DTM via an electron microscope showed that the tissue structure and its seminiferous tubules were well-preserved and completely depleted from cells. Further, an examination of the electron microscope indicated that the cells could sit well on the DTM after recellularization (Fig. 4).

### In vitro differentiation of SSCs

**Figure 5:**
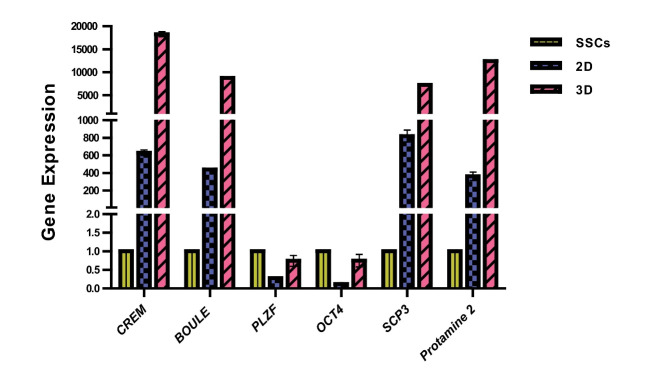
Expression of spermatogenesis genes )*OCT4,PLZF, SCP3, BOULE ,CREM ,Protamine2*), in cells cultured in DTM-based 3D substrate and in a 2D substrate analyzed by real-time PCR. The results are expressed as means ± SD.P<0.001.

**Figure 6:**
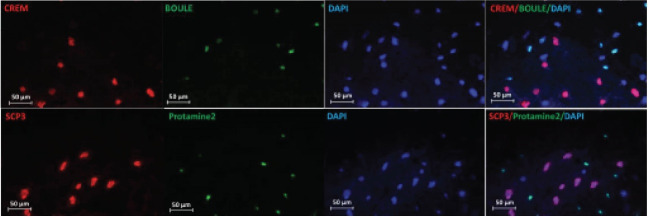
Immunocytochemistry staining was performed to evaluate the differentiation of the cells cultured on DTM for 6 weeks with spermatocyte markers (SCP3 & BOULE) and spermatid markers (CREM & Protamine2) Scale bars=50μm.

By extracting the RNA after six weeks of the cell culture on DTM and 2D substrate, the expression of per meiotic, meiotic and post meiotic genes were evaluated using qPCR method. Based on the results, the expression level of the pre meiotic, meiotic, and post meiotic genes was significantly higher on the DTM substrate (P≤0.001, Figure-5). In the sixth week since the initiation of SSCs culture and differentiation on DTM substrate, the expression of *BOULE, SCP3, CREM, Protamin2* was evaluated via immunocytochemistry. The results indicated the expression of spermatocytes and spermatids markers in cells cultured on the DTM substrate (Figure-6).

## Discussion

Long-term SCCs culturing under laboratory conditions can lead to the loss of unique properties of these cells. Therefore, achieving a culturing system, which can create the conditions of maximal resemblance to the body for the cells, can be effective in SCCs preservation, proliferation, and differentiation. Various factors can contribute to the maintenance and induction of SSCs including physical contact of these cells with adjacent cells and specific molecules present in the environment. Therefore, at each time interval, the adjacent cells, growth factors, and extracellular matrix compounds may modulate and regulate stem cell differentiation, division and apoptosis [16]. A large body of research was performed on SSCs culture and differentiation in three-dimensional substrates obtained from soft agar culture system (SACs), collagen gel (CG) & collagen+Matrigel (CGM), Methylcellulose Culture System (MCS), Poly (D, L-lactic-co-glycolic acid), or Poly-L-lactic acid PLGA (PLLA) [17-24]. The results confirmed the importance of 3D substrate in cell differentiation. However, due to the complex and unique structure of ECM, further studies are suggested for the decellularization of different tissues and their application to stem cells culture and differentiation [25-28]. In the present study, DTM was used as a three-dimensional substrate for SSCs culture and differentiation in order to investigate the effect of a natural matrix derived from testicular tissue possessing a unique ECM in SSCs differentiation. Maintaining ECM structure and composition plays an effective role on the behavior and migration of cultivated cells [29-32]. In this study, decellularized tissues were evaluated with histological staining and electron microscopy. Based on the results, the tissue structure and its seminiferous tubules were well-preserved and completely depleted from cells. SSCs proliferated in the laboratory for 4 weeks were examined by GFR α1 and PLZF antibodies via immunocytochemistry, and then they were cultured in a differentiation medium on DTM and 2D substrate for 6 weeks. In addition, the expression levels of pre meiotic, meiotic, and post meiotic genes were evaluated using the qPCR method. Based on the results, the expression rate of pre meiotic, meiotic and post meiotic genes are significantly higher in the DTM substrate than that of the 2D substrate. Further, the extension of culture duration contributed to the increased expression of the differentiation genes. The findings of the present study were consistent with those of the previous research on culture and differentiation in the 3D substrate [33-35]. The limitations of this study are the preparation of human testicular tissue and long cultivation of SSCs for reproduction and differentiation.

## Conclusion

The results of the present study indicated that the culture and differentiation of SSCs on a substrate, which could provide the natural structure of ECM, can prepare the suitable conditions for conducting more research on the spermatogenesis process in the in vitro medium, and contribute to the maintenance and treatment of male infertility.

## Acknowledgment

The authors thank all the staffs’ members of Tehran University of Medical Sciences and Human and Animal Cell Bank of Iranian Biological Resource Center who were involved in this project. This is an original article, which was supported by Tehran University of Medical Silences as a part of a Ph.D. student thesis (grant no. 35481).

## Conflict of interest

The authors declare that there is no conflict of interest.

**Table 1:** Primer sequences used for mRNA expression level of markers of different human spermatogenic stages.

**Gene name**	**Primers sequences (5’-3’)**	**Amplicon (bp)**
**GAPDH**	F: CATGAGAAGTATGACAACAGCCT R: AGTCCTTCCACGATACCAAAGT	113
**OCT4**	F: CTG GGT TGA TCC TCG GAC CT R: CAC AGA ACT CAT ACG GCG GG	128
**PLZF**	F: GTTGGAGTGAGATGAAGGAAGG R: AAGGTATGGGTGAAGGAAGGAGA	262
**SCP3**	F: TTGAAGATTTGGAGGGTGAAGT R: TCTGGTTAGTAGTTTTGAGAGAAG	129
**BOULE**	F: AAGGGTATGGTTTCGTCACTTTT R: GGACCGAAGTTACCTCTGGAG	249
**CREM**	F: ACACCACCTAGTATTGCTACCA R: GGATTGTTCCACCTTGGGCTAT	94
**Protamine2**	F: CAGTCTCACTATAGGCGCAG R: CTTAGTGCCTTCTGCATGTTCTC	163
